# Intrinsic Stability of Temporally Shifted Spike-Timing Dependent Plasticity

**DOI:** 10.1371/journal.pcbi.1000961

**Published:** 2010-11-04

**Authors:** Baktash Babadi, L. F. Abbott

**Affiliations:** 1 Center for Theoretical Neuroscience, Department of Neuroscience, Columbia University, New York, New York, United States of America; 2 Department of Physiology and Cellular Biophysics, Columbia University College of Physicians and Surgeons, New York, New York, United States of America; Université Paris Descartes, Centre National de la Recherche Scientifique, France

## Abstract

Spike-timing dependent plasticity (STDP), a widespread synaptic modification mechanism, is sensitive to correlations between presynaptic spike trains and it generates competition among synapses. However, STDP has an inherent instability because strong synapses are more likely to be strengthened than weak ones, causing them to grow in strength until some biophysical limit is reached. Through simulations and analytic calculations, we show that a small temporal shift in the STDP window that causes synchronous, or nearly synchronous, pre- and postsynaptic action potentials to induce long-term depression can stabilize synaptic strengths. Shifted STDP also stabilizes the postsynaptic firing rate and can implement both Hebbian and anti-Hebbian forms of competitive synaptic plasticity. Interestingly, the overall level of inhibition determines whether plasticity is Hebbian or anti-Hebbian. Even a random symmetric jitter of a few milliseconds in the STDP window can stabilize synaptic strengths while retaining these features. The same results hold for a shifted version of the more recent “triplet” model of STDP. Our results indicate that the detailed shape of the STDP window function near the transition from depression to potentiation is of the utmost importance in determining the consequences of STDP, suggesting that this region warrants further experimental study.

## Author Summary

Synaptic plasticity is believed to be a fundamental mechanism of learning and memory. In spike-timing dependent synaptic plasticity (STDP), the temporal order of pre- and postsynaptic spiking across a synapse determines whether it is strengthened or weakened. STDP can induce competition between the different inputs synapsing onto a neuron, which is crucial for the formation of functional neuronal circuits. However, strong synaptic competition is often incompatible with inherent synaptic stability. Synaptic modification by STDP is controlled by a so-called temporal window function that determines how synaptic modification depends on spike timing. We show that a small shift, or random jitter, in the conventional temporal window function used for STDP that is compatible with the underlying molecular kinetics of STDP, can both stabilize synapses and maintain competition. The outcome of the competition is determined by the level of inhibitory input to the postsynaptic neuron. We conclude that the detailed shape of the temporal window function is critical in determining the functional consequences of STDP and thus deserves further experimental study.

## Introduction

Hebbian synaptic plasticity can effectively organize neural circuits in functionally useful ways, but only when implemented in a manner that induces competition among synapses [1]. Spike-timing dependent synaptic plasticity (STDP), which has been observed in a wide variety of preparations (see [2] for a review), appears to provide such an implementation by forcing synapses to compete for control of the timing of postsynaptic action potentials while being strengthened or weakened. In STDP, a synapse is potentiated when a presynaptic action potential precedes a postsynaptic spike, and depressed otherwise (see [3] for a review). STDP has been shown to induce a competitive form of Hebbian plasticity that is useful for a variety of neuro-computational problems (see [4] for a review). However, this form of STDP has an inherent instability in that strong synapses get stronger and weak synapses get weaker. This instability can be tamed by biophysical limitations on synaptic strengths, resulting in a U-shaped distribution of synaptic efficacies [5]. Nevertheless, it is interesting to examine models that do not require such constraints for stabilization and that generate unimodal distributions of synaptic strengths resembling those measured in cultured and cortical networks [6]–[8].

Synaptic competition and synaptic stability (meaning that synapses reach a stable equilibrium distribution independent of bounds on their strengths) are desirable but conflicting features of Hebbian synaptic plasticity. For example, the instability of STDP mentioned in the previous paragraph can be eliminated by introducing strength-dependent modification [9], [10], but at the expense of eliminating synaptic competition. By interpolating between stable and unstable models of STDP, it is possible to obtain both synaptic competition and stability, but over a limited parameter range [11]. Here we propose an alternative solution inspired by the slow kinetics of NMDA receptors. We show that STDP can be stabilized if the boundary separating potentiation and depression does not occur for simultaneous pre- and postsynaptic spikes, but rather for spikes separated by a small time interval. Through simulation as well as by solving the Fokker-Planck equation governing the distribution of synaptic strengths, we show that any positive shift of the STDP window can stabilize the distribution of synaptic strengths while preserving synaptic competition. These properties also hold for a multi-spike STDP rule in which triplets of pre- and postsynaptic spikes are the key events in determining the synaptic change [12], as opposed to pair-based STDP in which pairs of pre- and postsynaptic spikes govern the plasticity process. Moreover, our simulations show that even a random symmetric jitter of a few milliseconds in the STDP window can stabilize synaptic strengths while retaining these features.

## Results

To study the effects of STDP on synaptic strengths, we simulated a single spiking neuron that receives excitatory and inhibitory presynaptic spike trains with Poisson statistics at rates 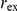 and 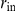, respectively (Methods). The strengths of the excitatory synapses, denoted by 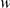, change due to STDP, while the strengths of the inhibitory synapses remain constant. We first consider the pair-based model of STDP. A more complicated multi-spike model will be studied afterward. In the pair-based model the change in synaptic strength, 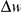, induced by a pair of pre- and postsynaptic action potentials with time difference 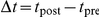 is determined by
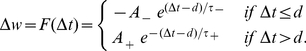
(1)The parameters 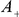 and 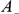, both positive, determine the maximum amount of synaptic potentiation and depression, respectively. We define synaptic strengths in units of membrane potential depolarization (mV), so 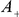 and 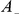 have mV units as well (Methods). The time constants 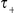 and 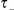 determine the temporal extent of the STDP window for potentiation and depression. The parameter 
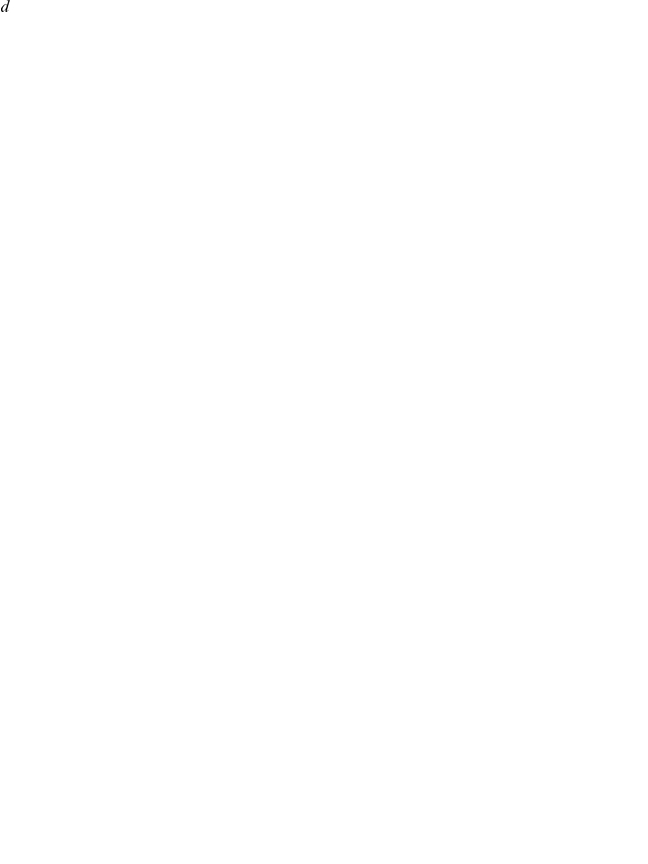
, also positive, introduces a shift in the STDP window such that even in cases where a presynaptic action potential precedes the postsynaptic spike by a short interval (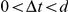), the corresponding synapse gets depressed. Note that we recover conventional pair-based STDP by setting 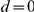. Further details of the synaptic modification procedure appear in the Methods, and the numerical values of the STDP parameters are given in Table 1. An important feature of the pair-based model we use is that STDP arises solely from pairs of pre- and postsynaptic spikes that are nearest neighbors in time, in agreement with experimental results [13]. Specifically, each postsynaptic action potential can only potentiate a synapses on the basis of the interval to the presynaptic spike immediately preceding it, and each presynaptic action potential can only depress a synapses on the basis of the timing interval to the immediately preceding postsynaptic spike. This assumption is important for the results we obtain using the pair-based STDP model, as discussed below.

**Table 1:** Neuronal, synaptic, and plasticity parameters.

Parameter	Symbol	Default value
Membrane time constant	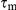	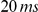
Spiking threshold	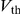	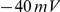
Resting membrane potential	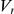	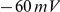
Maximum potentiation amplitude	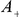	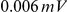
Maximum depression amplitude	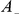	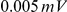
Potentiation time constant	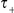	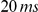
Depression time constant	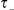	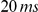
Window shift	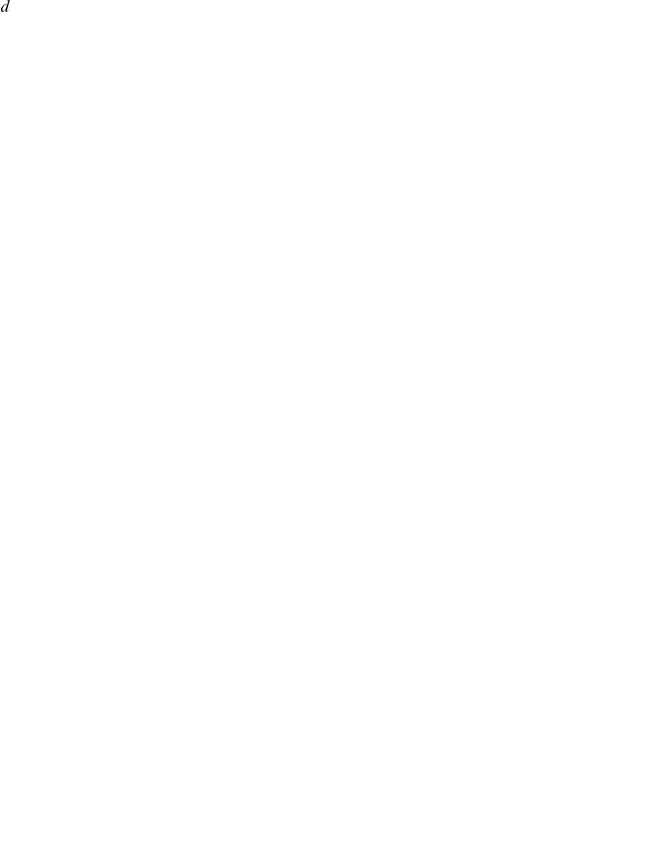	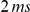
Synaptic time constant	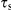	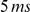
Number of excitatory synapses	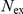	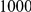
Number of inhibitory synapses	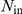	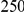
Inhibitory synaptic strength	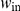	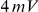
Excitatory input rate	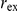	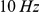
Inhibitory input rate	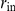	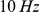

### Stability of synaptic strengths

**Figure 1:**
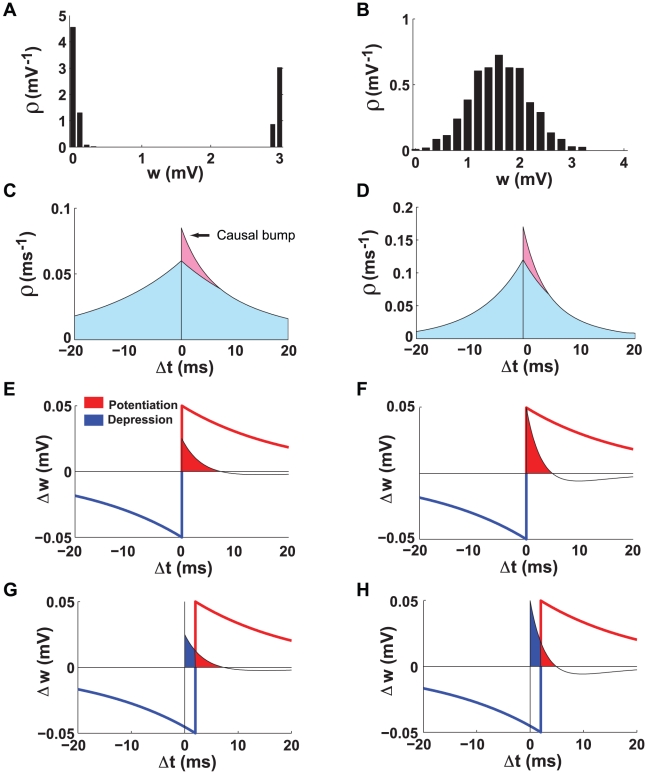
Comparison of unshifted and shifted STDP. **A**. The U-shaped steady-state distribution of synaptic strengths for conventional unshifted STDP. **B**. The unimodal steady-state distribution of synaptic strengths for shifted STDP (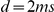). **C**. The probability density of pairing intervals for presynaptic and postsynaptic spike trains. The blue area is the symmetric acausal contribution, and the pink area is the additional causal bump arising from postsynaptic spikes induced by the presynaptic input. **D**. Same as C, but for a stronger synapse. The causal bump is larger and closer to 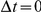. **E**. The causal bump superimposed on the unshifted STDP window. The potentiation part of the STDP curve is red and the depression part blue. The causal bump falls entirely within the potentiation domain (red shading). **F**. Same as E, but for a stronger synapse. The causal bump still falls within the potentiation region. **G**. Same as E, but for shifted STDP. Part of the causal bump falls into the depression region (blue shading). **H**. Same as G, but for a stronger synapse. More of the causal bump falls into the depression region.

With conventional, unshifted STDP (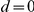), synaptic strengths grow or shrink indefinitely unless limits are imposed. These limits produce a U-shaped distribution of synaptic strengths (figure 1A, [5]). However, if we introduce a 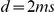 shift into the STDP window, the steady-state distribution of synaptic strengths is unimodal and stable even when no limits are imposed (figure 1B). Why does this occur?

The total effect of a sequence of pre- and postsynaptic action potentials on the strength of a synapse can be computed by multiplying the STDP window function by the probability of a spike pair appearing with time difference 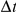 and then integrating over all values of 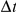. If we assume Poisson spike trains and ignore the effects of the synapse, the probability distribution of nearest-neighbor pre-post pairs is an exponentially decaying function of the magnitude of the interval between them (figure 1C). The decay rate of this exponential is equal to the sum of the pre- and postsynaptic firing rates (Methods). The presence of a synapse induces an additional contribution to this distribution for small positive 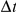 arising from postsynaptic spikes induced by the synaptic input (figure 1C). The size of this “causal bump” is proportional to the probability of a presynaptic action potential evoking a postsynaptic response, and hence to the strength of the synapse. The stronger the synapse, the larger the bump. In addition, because the postsynaptic spike latency is shorter for stronger synapses, the bump moves closer to 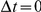 as the synaptic strength increases (figure 1D). These features of the pre-post interval distribution are crucial for our analyses.

**Figure 2:**
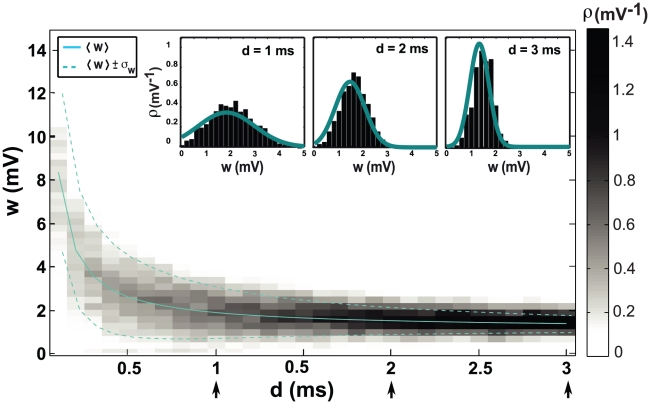
Shifted STDP stabilizes the distribution of synaptic strengths. The horizontal axis is the value of the shift, the vertical axis is the synaptic strength and the gray level is the probability density of strengths, obtained by simulation. Solid line is the analytically calculated mean and dashed lines show the analytically calculated standard deviation around the mean. Insets show the distribution of synaptic strengths for different values of the shift. Solid curves are analytically calculated distributions. The arrows at the bottom of the horizontal axis of the main plot show the shift values corresponding to the insets.

When there is no shift in the STDP window, the causal bump falls entirely within the potentiation domain (figure 1E), which is why synaptic strengths grow until something else stops them (figure 1F). When the STDP window is shifted, part of the causal bump falls into the region where depression occurs (figure 1G). Furthermore as the synapse gets stronger, a larger portion of the causal bump falls into the depression domain, both because the causal bump gets bigger and because it moves closer to 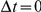 (figure 1H). This prevents further growth of the synaptic strength and explains why a shift stabilizes synaptic growth through STDP. Stabilization of synaptic weights occurs for any positive value of the delay (
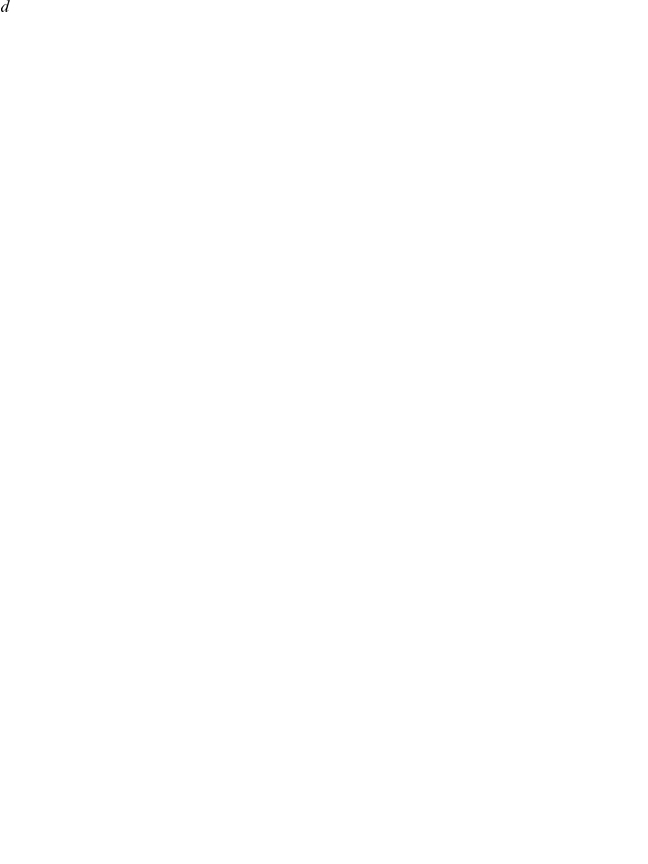
), but larger delays result in lower mean values and sharper distributions for the weights (figure 2).

**Figure 3:**
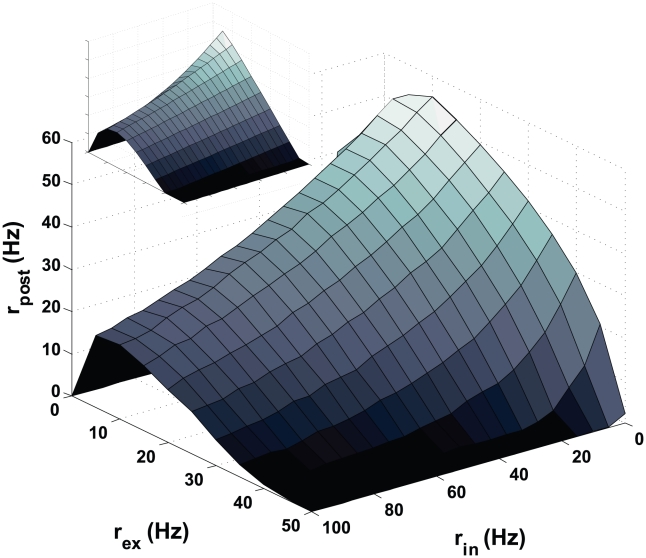
The steady-state postsynaptic firing rate. The steady state firing rate is plotted as a function of the input rates for excitation and inhibition. The inset shows the corresponding analytic result.

For a more quantitative evaluation of shifted STDP, we computed the steady-state solution of the Fokker-Planck equation governing the distribution of synaptic strengths [14]–[16] (Methods). With a few reasonable approximations and ignoring any limits or bounds, the steady-state distribution of synaptic strengths has the form of a gamma distribution,
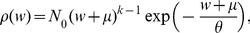
(2)where 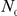 is a normalization constant and 
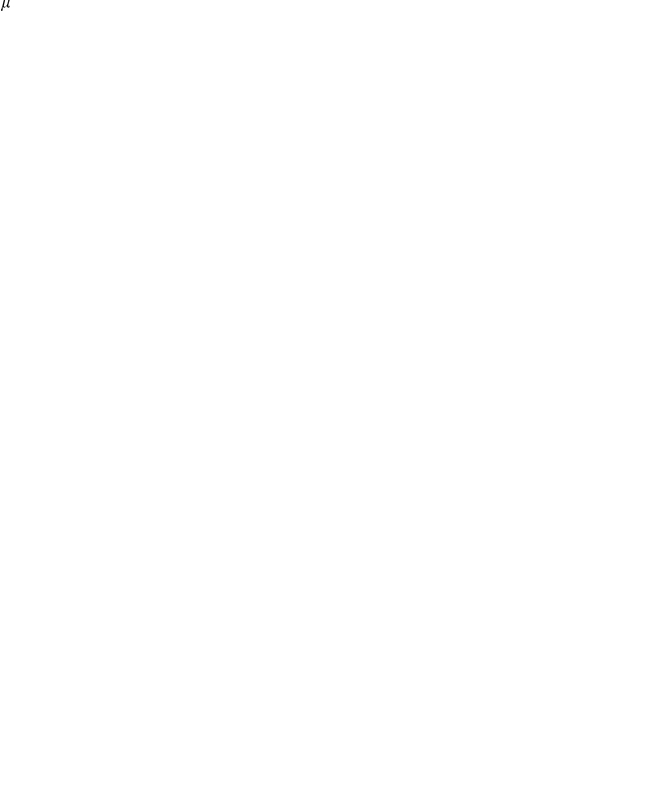
, 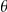 and 
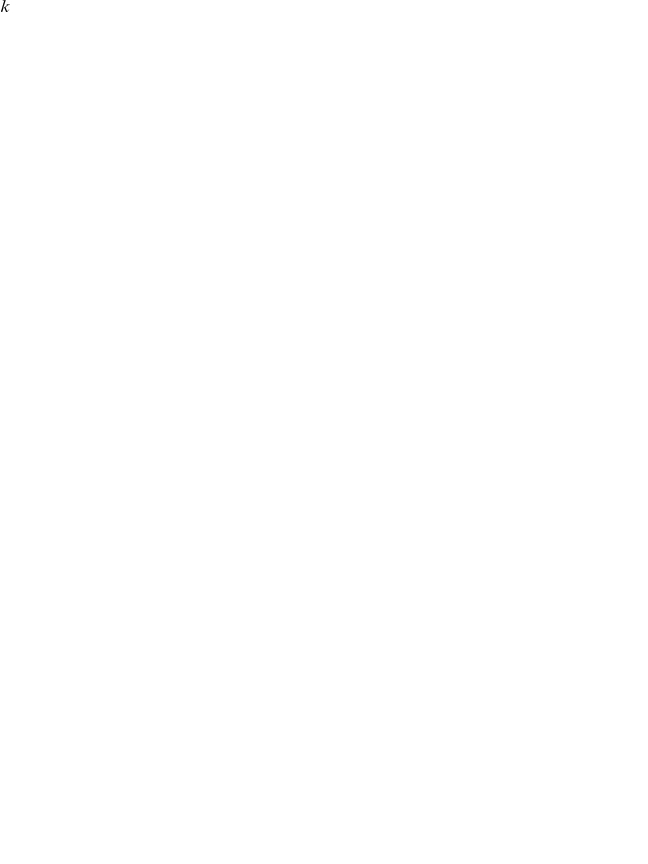
 are computed parameters. If either 
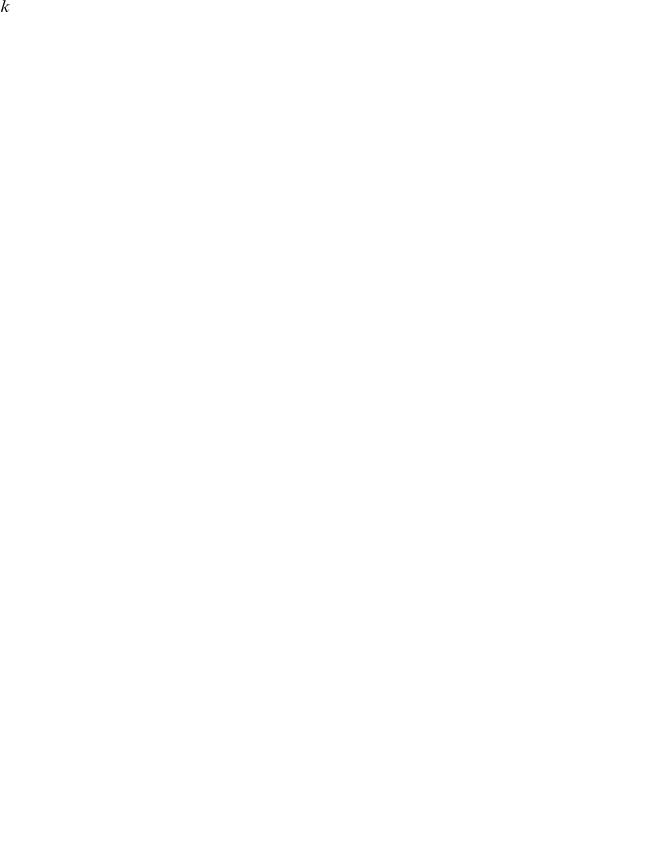
 or 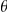 is negative, this distribution cannot be normalized, implying unstable synaptic strengths. The calculations indicate that 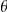 is positive for any positive shift (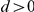, Methods). Positivity of 
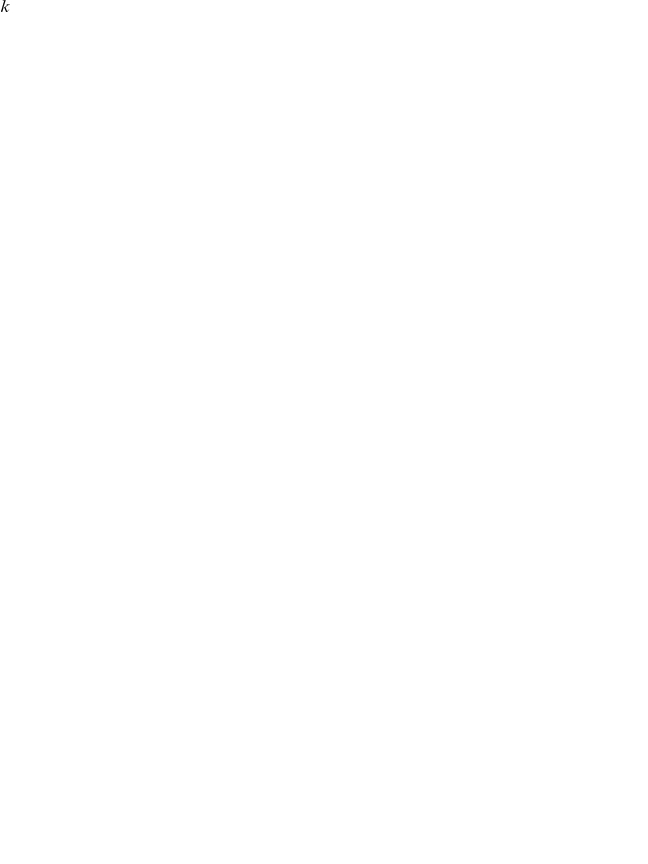
 requires that 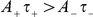. Note that this is opposite to the condition required of conventional, unshifted STDP (see for example [5]). Because it is easier to do the analytic calculations without imposing strict boundary conditions on the synaptic strengths, the analytic formula sometimes includes a small probability for negative strength synapses, which is not allowed in the simulations. Other than this small discrepancy, the agreement between the analytic distribution and the simulation results is good (figures 2 & 3). In what follows, 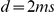, 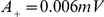, 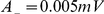, and 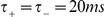, unless stated otherwise.

### Steady-state firing rate

STDP has an interesting regulatory effect on the steady-state firing rate of a neuron [5], [15]. With unshifted STDP, this is a buffering effect making the steady-state postsynaptic firing rate relatively insensitive to the firing rates of excitatory and inhibitory inputs. Shifted STDP also buffers the postsynaptic firing rate, but the residual dependence on the presynaptic rates displays an interesting effect. Although the steady-state firing rate decreases when the inhibitory input rates are increased, it has a surprising non-monotonic dependence on the rates of excitatory inputs (figure 3).

The stabilization of synaptic strengths discussed in the previous section arises from the change of size and shape of the causal bump seen in figure 1C & D. Buffering of the steady-state postsynaptic firing rate is affected primarily by the shape of the symmetric, non-causal component of the spike-timing probability. As mentioned previously, this component falls off exponentially, for either positive or negative spike-timing differences, at a rate given by the sum of the presynaptic and postsynaptic firing rates (Methods). If this sum grows, the acausal part of the distribution gets more peaked near zero, bringing more spike pairs into the region of the STDP window where the shift leads to synaptic depression. The resulting reduction in synaptic strength then lowers the postsynaptic firing rate. This form of buffering would not be present if all spike pairs, rather than only nearest-neighbor pairs, were involved in STDP. If we allowed all spike pairs to induce synaptic plasticity the relevant symmetric, non-causal distribution would be flat, rather than exponentially decaying. In this case, there is no analogous stabilization and, in fact, postsynaptic rates slowly rise, making the plasticity unstable, even with shifted STDP. This is why we require shifted STDP to be based only on nearest-neighbor spike pairs.

In general, we expect the firing rate of a neuron to increase when its excitatory inputs fire more rapidly, and this is exactly what occurs for excitatory input rates below about 10 Hz in figure 3. However, for excitatory input rates higher than this, the steady-state (after STDP has equilibrated) postsynaptic firing rate decreases. This occurs for the reason outlined in the previous paragraph. Increasing the presynaptic rate causes the acausal distribution to sharpen and induces synaptic depression. This slows the postsynaptic rate, broadening the acausal distribution until the spike intervals in the delay region are sufficiently reduced in number. This is what causes the steady-state postsynaptic firing rate to drop when the excitatory presynaptic rates are raised to high levels.

Shifted STDP also has a buffering property on changes in the inhibitory input rate. In presence of strong inhibitory input, the postsynaptic firing rate falls. This broadens the acausal part of the spike-pair distribution, lowering the chance for pairs to fall into the depression domain caused by the shift and, thus, resulting in more potentiation. However, in this case, the effect is not strong enough to overcome the expected tendency of the postsynaptic rate to be suppressed by inhibition (figure 3).

### Synaptic competition

Hebbian plasticity in general and STDP in particular allows neurons to become selective to correlated subsets of their inputs, but this requires synaptic competition [1]. We call synaptic plasticity “competitive” if correlating a subset of synaptic inputs causes both that set and the remaining synapses to change their strengths in an opposing manner, so than either the correlated or the uncorrelated set of synapses gains control of the postsynaptic firing (see for example [11]). In particular, if STDP is competitive, the strengths of either the correlated or uncorrelated subgroup of synapses should cluster near zero. To determine whether the necessary competition exists with shifted STDP, we imposed pairwise correlations with a coefficient of 0.2 on one half of the incoming excitatory spike trains while leaving the other half uncorrelated (Methods). With unshifted STDP, this arrangement induces a competition that correlated synapses always win [5]. In other words, the synapses receiving correlated input become stronger and those receiving uncorrelated input get weaker.

**Figure 4:**
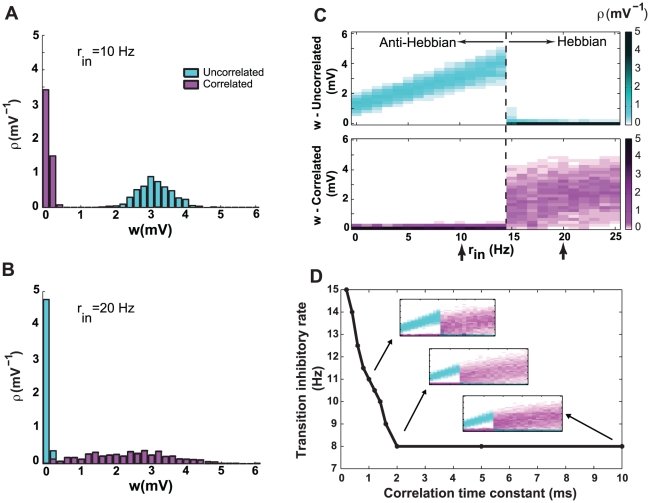
Synaptic competition through shifted STDP. Cyan color indicates synapses with uncorrelated inputs, and magenta indicates correlated inputs. The rate of excitatory input is fixed at 10 Hz, and the correlation coefficient is 0.2 for correlated input spike trains. **A**. Steady-state distribution of synaptic strengths for an inhibitory rate of 10 Hz. Uncorrelated synapses become stronger than correlated. **B**. Steady-state distribution of synaptic strengths for an inhibitory rate of 20 Hz. Correlated synapses now become stronger than uncorrelated. **C**. Distributions of strengths for synapses receiving uncorrelated (top) and correlated (bottom) inputs as a function of the inhibitory input rate. The color level indicates the probability density of strengths. A transition from anti-Hebbian to Hebbian competition occurs at an inhibitory input rate of 14 Hz (dotted line). Arrows indicate the parameters for panels A and B. **D**. The transitional inhibitory rate as a function of correlation time constant. The transition takes place at lower inhibitory rates as the correlation time constant increases up to 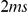, then remains constant at 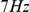 for higher values. The insets show the full distribution of correlated and uncorrelated synaptic strengths as in C, for correlation time constants of 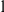, 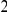 and 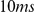.

Interestingly, with shifted STDP the outcome of the competition depends on the rate of inhibitory input to the neuron. When the rate of inhibitory input is 10 Hz for the parameters we use, the synapses receiving correlated spikes end up weaker than the synapses receiving uncorrelated spikes (figure 4A). This behavior is “anti-Hebbian” in that it is opposite to what is expected from normal Hebbian modification. However, when the rate of the inhibitory inputs is increased to 20 Hz, we obtain the usual Hebbian result in which correlated synapses win the competition and become stronger than uncorrelated synapses (figure 4B). Results obtained over a range of inhibitory input rates show a transition from anti-Hebbian to Hebbian modification (figure 4C). Choosing other values for the correlation coefficient within a range from 0.1 to 0.9 yielded qualitatively similar results. Competition also occurs between two correlated subgroups with different correlation coefficients, with the more correlated synapses dominating over the less correlated ones in the Hebbian (high inhibition) case and vice versa in the anti-Hebbian (low inhibition) mode. If the correlation coefficients for the two groups are the same, no competition takes place.

These results were obtained using spike trains with zero time-lag correlations, meaning that for any two correlated spike trains, a subset of spikes is perfectly synchronous. More realistic spike correlations can be generated by including a small random jitter in the timing of the synchronous spikes. The mean of this jitter determines the correlation time constant. Breaking perfect synchrony does not change the above results qualitatively. However, the rate of inhibitory input needed to transition from anti-Hebbian to Hebbian competition is sensitive to the correlation time constant (figure 4D). When the correlation time constant increases, the inhibitory rate at the transition decreases, until the correlation time constant becomes greater than the shift of the STDP window (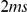). Further increase in the correlation time constant does not lead to any more lowering of the transitional inhibitory rate (figure 4D).

The dependence of the outcome of synaptic competition on the level of inhibitory input can be explained by evaluating the effect of inhibition on the firing regime of the postsynaptic neuron. When the inhibitory input to a neuron is low, it operates in a “mean-driven” regime, meaning that the time-averaged “free-running” membrane potential (that is, the membrane potential if the spike generation mechanism is turned off) is above the firing threshold [17]. In the mean-driven regime, integrate-and-fire neurons spike regularly, so the coefficient of variation of the inter-spike-intervals (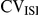), which is a measure of the irregularity of firing, is small [18]. On the other hand, when the inhibitory input to the neuron is high, the mean membrane potential is below the firing threshold. In this case, large deviations in the membrane potential from its mean are required to make the neuron fire, and the neuron is said to be in the “fluctuation-driven” regime [17]–[19]. This makes firing times irregular, resulting in a larger 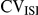.

**Figure 5:**
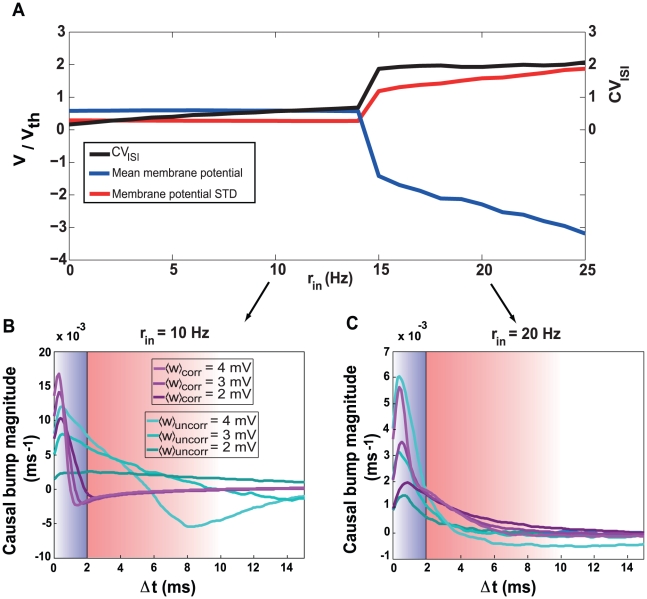
The effect of the inhibitory input on synaptic competition. **A**. Transition from mean-driven to fluctuation-driven firing regimes when the rate of the inhibitory input is increased. The black curve is the coefficient of variation of postsynaptic interspike intervals (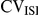), the blue curve is the mean free-running membrane potential in units of the spiking threshold, and the red curve is the standard deviation of the membrane potential in the same units. For inhibitory input rates greater than 14 Hz, there is an abrupt switch from the mean-driven to the fluctuation-driven regime, corresponding to the transition from anti-Hebbian to Hebbian competition (figure 4). **B**. Postsynaptic causal bumps due to uncorrelated (cyan) and correlated (magenta) input spikes for different mean synaptic strengths (shading) when the inhibitory input rate is 10 Hz. The blue area shows the depression domain and the red area is the potentiation domain. Note that the correlated causal bumps (magenta) fall almost entirely into the depression domain (blue shading) in this case, so the correlated synapses lose the competition. **C**. Same as panel b, but for an inhibitory input rate of 20 Hz. Note the heavy tail of the correlated causal bumps (magenta), which extend into the potentiation domain of the STDP window. These curves were obtained by numerical simulations, changing the mean of the steady-state distribution of correlated or uncorrelated synapses to the desired value for each curve. Because the correlated synapses arrive in unison, their causal bump is the aggregate effect of all of their spikes. To show the contribution of individual correlated spikes, comparable to that of the uncorrelated ones, we therefore normalized the magnitude of the causal bump of the correlated synapses by their average cluster size (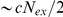).

The model neuron we study traverses these regimes as the firing rate of its inhibitory inputs is varied (figure 5A). When the inhibitory input is small, the neuron operates in the mean-driven regime, with its mean free-running membrane potential above threshold and a small 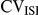. When the inhibitory input rates increase beyond 14 Hz, the neuron suddenly switches to a fluctuation-driven regime in which the mean membrane potential is below threshold and 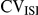 is large. The transition between the mean-driven and fluctuation-driven regimes occurs exactly where synaptic competition switches from being anti-Hebbian to Hebbian (compare figure 4C with 5A). Thus, the key feature determining whether plasticity is anti-Hebbian or Hebbian is whether the postsynaptic neuron is in a mean-driven or fluctuation-driven state.

Recall that the causal bump is the excess probability of postsynaptic firing caused by an incoming input spike. As mentioned previously, the effect of shifted STDP on the distribution of synaptic strengths can be explained by considering the shape of the postsynaptic causal bump in relation to the STDP temporal window. When the postsynaptic neuron is in the mean-driven regime, the membrane potential rises rapidly to the threshold. As a result, presynaptic action potentials can only enhance postsynaptic firing if they occur during a relatively short time-interval prior to the postsynaptic spike. This means that the causal bump decays rapidly for longer intervals. The causal bump also has a higher amplitude and decays more rapidly for stronger synapses (figure 5B). Furthermore, the causal bump due to correlated inputs is even narrower and sharper (and more inside the depression region) than the bump due to uncorrelated inputs (figure 5B, magenta traces), because correlated spikes are more likely to induce a postsynaptic spike rapidly when they occur in unison. As a result, the uncorrelated synapses win the synaptic competition when the level of inhibition is low.

When the postsynaptic neuron fires in the fluctuation-driven regime, the membrane potential spends a considerable time near but below the firing threshold before spiking. As a result, presynaptic input can affect postsynaptic firing over a longer time interval than in the mean-driven regime. This makes the causal bump broader than in the mean-driven case (figure 5C). Furthermore, the causal bump is even broader for correlated than for uncorrelated inputs because the simultaneous arrival of correlated spikes generates a stronger depolarization transient that makes it possible for subsequent weaker inputs to push the postsynaptic neuron above threshold over a longer time interval. This gives the causal bump for the correlated inputs a long tail that extends well into the potentiation domain of the STDP window (figure 5C, magenta traces), allowing them to win the competition in this case.

The transition from the mean-driven to the fluctuation-driven regime and correspondingly from anti-Hebbian to Hebbian competition is quite abrupt. This may be due to the interplay between the correlated inputs and the firing mode of the neuron. Correlated inputs increase membrane potential fluctuations and spiking irregularity [20]. Therefore, within the context of shifted STDP, there is positive feedback between the fluctuation-driven regime and the dominance of correlated inputs. As the neuron transitions to the fluctuation-driven regime through increased inhibition, the correlated synapses start to strengthen more than the uncorrected ones which, in turn, increases the fluctuations of the membrane potential and pushes the neuron further into the fluctuation-driven regime. This positive feedback continues until the correlated synapses dominate over the uncorrelated ones and the neuron falls completely into the fluctuation-driven mode.

### Jittered STDP window

**Figure 6:**
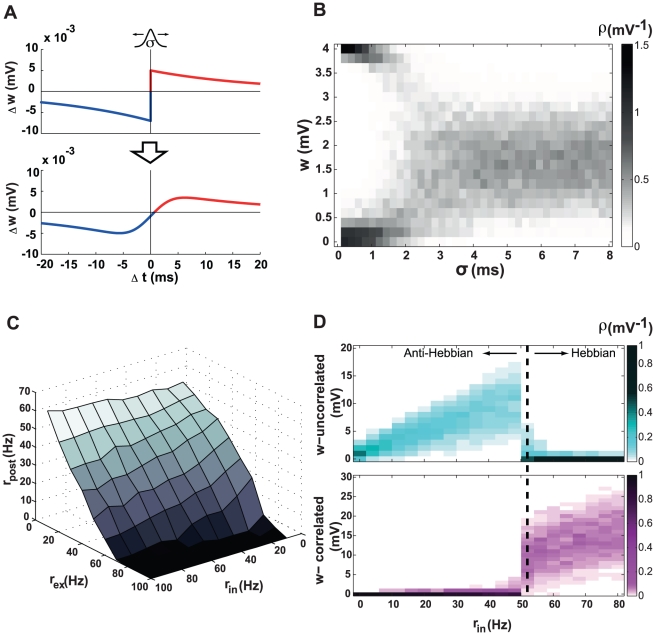
Jittered STDP. **A**. A random symmetric jitter of the unshifted STDP window (top) results in an effective window function (bottom) in which depression is dominant for short positive pairing intervals (blue shading). **B**. Jittered STDP stabilizes the distribution of synaptic weights. The horizontal axis is the standard deviation of the jitter (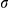), the vertical axis is synaptic strength and the gray level indicates the probability density of strengths. For jitters smaller than 2 ms the distribution is bimodal, but for larger jitters it is stable and unimodal. **C**. The steady-state firing rate of the postsynaptic neuron as a function of the excitatory and inhibitory input rates when the jitter is 3 ms. **D**. Jittered STDP (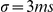) implements both Hebbian and anti-Hebbian competition. As in figure 4, the top panel shows the distribution of uncorrelated synapses (cyan) and the bottom panel shows the distribution of correlated synapses (magenta), both as functions of the inhibitory input rate. The transition from anti-Hebbian to Hebbian competition occurs when the inhibitory input rate is about 50 Hz in this case.

It is not necessary to introduce an explicit shift into the STDP window to assure stability. Any mechanism that causes depression to dominate over potentiation for short positive pairing intervals will have the same qualitative effect. One such mechanism is a symmetric random jitter introduced into an unshifted STDP window that has 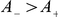. By jitter we mean that the time 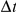 used to determine the effect of STDP for any given pair of pre- and postsynaptic spikes, is not simply the difference between the times of their occurrence, but instead a random term is added. In other words, 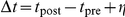, where 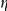 is a random variable drawn from a distribution with zero mean and a certain variance (we use a Gaussian distribution). Although the STDP window has no explicit shift in this case (figure 6A, top), the effective window obtained by averaging over the symmetric random jitter (figure 6A, bottom), exhibits the required feature that depression occurs for small positive pairing intervals.

Simulations show that jittered STDP has all the qualitative properties of shifted STDP, although the maximum depression must be set to be greater rather than the maximum potentiation (we take 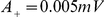 and 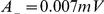, although see [21]). To keep 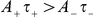, as required for stability, the time constant of potentiation must be larger than that of depression (we take 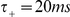 and 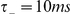). If the standard deviation of the jitter is less than 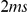, the steady-state distribution of synaptic weights is not inherently stable and we obtain a U-shaped distribution of synaptic strengths (figure 6B). However, for larger standard deviations of the jitter, the steady-state distribution is stable and unimodal as in the case of shifted STDP (figure 6B). Other features of shifted STDP are also reproduced. The steady-state firing rate of the postsynaptic neuron decreases when the rate of presynaptic input increases (figure 6C), and either anti-Hebbian or Hebbian competition occurs depending on the rate of inhibitory input to the neuron (figure 6D).

### Shifted STDP with multi-spike interactions

A pair-based STDP model cannot account for all experimentally observed spike-timing dependent synaptic modifications. When bursts of spikes are induced in the pre- and postsynaptic neurons, frequency dependence is observed for both pre-after-post and post-after-pre pairings. The magnitude of LTP, but not LTD, increases with burst frequency and, at high burst frequency, LTP is induced regardless of the ordering of the pre- and postsynaptic spikes [13], [22]. Similarly, the dependence of synaptic modification on triplets and quadruplets of spikes cannot be fully explained by pair-based STDP models [23], [24]. Recent results from experiments that used complex spike patterns have led to STDP models that take into account interactions between multiple pre- and postsynaptic spikes. The details of the multi-spike interactions vary among different models. In the “suppression model” the plasticity-inducing effect of each pre- or postsynaptic spike is suppressed by the preceding spikes in the same neuron [22], [23]. In the “triplet model”, in addition to the effect of neighboring pre-post pairings there is an extra depression exerted by the preceding presynaptic spikes and an extra potentiation by the preceding postsynaptic spikes [12]. The triplet model can account for most of the observed synaptic modifications induced by complex spike patterns [12], including the dependency of plasticity on burst frequency [13] and triplet effects in hippocampal culture [24] where pre-post-pre ensembles with the same timing difference lead to insignificant changes but post-pre-post ensemble induces a strong potentiation of synapse. The triplet model can also be mapped to a Bienenstock-Cooper-Munro learning rule [25], which has several interesting functional properties. Nevertheless, the triplet model suffers from the same instability as pair-based STDP. Therefore, we examine the effect of introducing a shift into this model.

In the triplet model, a “2 pre/1 post” ensemble of spikes exerts an extra depression (the triplet depression) in addition to the usual pre-post pairing effect. The triplet depression has its maximum value 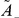 immediately after the first presynaptic spike and decays exponentially as a function of the interval between the two presynaptic spikes, with time constant 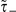. Similarly, a “1 pre/2 post” ensemble of spikes exerts an extra potentiation (the triplet potentiation) with the maximum value 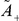 and decay time constant 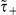 (see Methods for details). The value of these triplet parameters vary in different neuronal preparations [12]. Here, we set the time constants 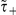 and 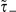 to 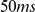 and examine the model with a range of parameters 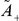 and 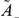. The window of the pre-post pairing is shifted by 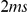 as before and all other parameters are the same as in Table 1.

**Figure 7:**
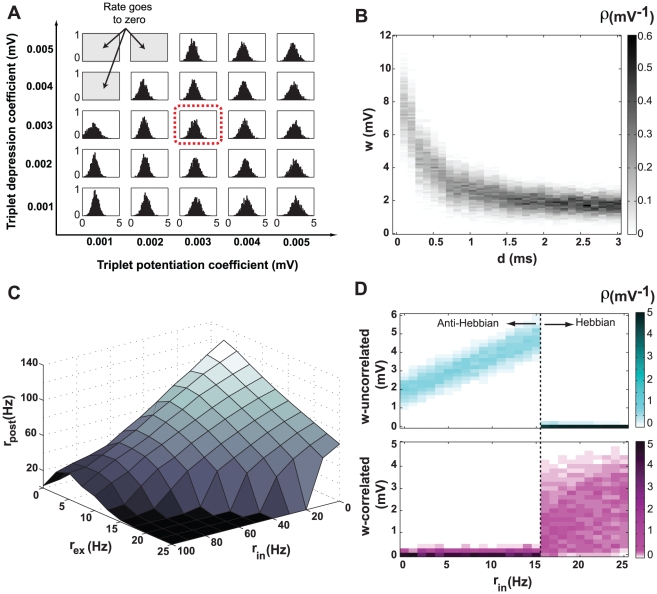
The shifted triplet model. **A**. The final distribution of weights for different values of maximum triplet potentiation (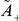) and depression (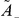). Except for very high depression values, the distribution is unimodal and stable. We used the representative value of 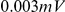 for both 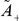
and 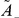 (red dotted box) for the remaining results in this figure. **B**. The shift stabilizes the distribution of synaptic weights. The horizontal axis is the value of the shift, the vertical axis is the synaptic strength, and the gray level is the probability density of the strengths (as in figure 2), obtained by simulation. **C**. The steady-state firing rate of the postsynaptic neuron as a function of the excitatory and inhibitory input rates. **D**. The shift in the triplet model can implement both Hebbian and anti-Hebbian competition. As in figure 4, the top panel shows the distribution of the uncorrelated synapses (cyan) and the bottom panel shows the distribution of the correlated ones (magenta), as a function of the inhibitory input rate. The transition from anti-Hebbian to Hebbian competition occurs at an inhibitory input rate of 16 Hz.

Simulations show that the final distribution of weights is stable and unimodal (figure 7A) using the triplet model, unless the triplet depression is extremely high, which causes the firing rate of the postsynaptic neuron to go to zero, terminating plasticity. After finding that the shifted STDP in the triplet model stabilizes weights for a wide range of parameters, we set 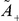 and 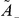 to 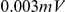 and examined other properties of shifted STDP in this model. Further simulations showed that shifted STDP within the framework of the triplet model has all the qualitative properties of the shifted pair-based STDP model. A shift as low as 0.1 milliseconds is sufficient to stabilize the weights, with larger delays resulting in lower mean values and sharper distributions for the weights (figure 7B). The steady-state firing rate of the postsynaptic neuron decreases when the rate of the excitatory and/or inhibitory presynaptic input increases (figure 7C). Finally, either anti-Hebbian or Hebbian competition occurs depending on the rate of inhibitory input to the neuron (figure 7D).

## Discussion

We have shown that a slight shift in the effective STDP temporal window, such that postsynaptic spikes occurring shortly after presynaptic action potentials cause synaptic depression, can stabilize the distribution of synaptic strengths without loss of competition, both in pair-based and triplet-based models. The shift can be explicitly implemented in the STDP window or achieved by other means such as a symmetric spike-by-spike random jitter. In fact, any mechanism that causes synaptic depression for small but causal (positive by our convention) pre-post spike intervals should lead to the stabilization and other effects we report. What biophysical mechanisms could cause this to occur?

The sharp transition between depression and potentiation in STDP appears to be due to the abrupt onset of long-term potentiation [26], [27]. It is believed that the Ca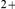 influx through NMDA receptors is responsible for this potentiation [28] and that the abrupt onset arises because the NMDA channel be in an open but blocked configuration before subsequent depolarization removes the Mg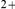 block [29]. To assure a large Ca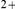 influx and subsequent potentiation, it seems reasonable to assume that the depolarization that removes the Mg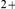 block should occur near the peak of the NMDA activation. The Mg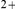 removal by postsynaptic depolarization is extremely rapid [30] but the NMDA activation has a finite rise time, so the peak of NMDA activation occurs a few milliseconds after the arrival of the presynaptic spike [31]. Therefore, it seems likely that the maximum potentiation should occur when the presynaptic spike precedes the postsynaptic action potential by several milliseconds, and that depression could result from timing differences shorter than this. Thus, the biophysics of the NMDA receptor appears to support the idea of a temporal shift in the STDP window. The shape of the STDP window has been inferred from models of NMDA receptor kinetics and back-propagating action potentials [32], [33]. However, the millisecond timing of the transition from depression to potentiation was not investigated systematically, because its significance was not evident at that time. Nevertheless, in some parameterizations of such models a small depression domain for short positive pairing intervals has been reported [33].

Typically in electrophysiological recordings, action potentials are measured at the soma, but what matters for STDP is the timing of the events at the synapse. More precisely, the timing of the postsynaptic EPSP and that of the backpropagating action potential to the synapse control plasticity. Transmission delays may have their own interesting computational properties. For example, it has been shown that STDP in the presence of axonal transmission delays can have a desynchronizing effect on population bursts and a synchronizing effect on random spiking in a recurrent network [34]. The transmission delay of the EPSP to the soma and that of the backpropagating action potential subtract from the delay we need for shifted STDP. For distal synapses where these delays are longer, there may be a higher probability that the causal bump falls out of the depression domain caused by the shift. This might be a mechanism for counterbalancing the attenuation of the EPSPs arising from distal dendrites [35], [36] along with other proposed mechanisms [37], [38]. It may explain the enhancement of LTD reported in studies of STDP at distal sites [21], [39], [40]. If the delay becomes longer than the shift for very distal synapses, other mechanisms such as limits on synaptic strength must serve to stabilize STDP. Finally, if the speed of backpropagating action potential can be increased through modification of voltage-dependent conductances, the model predicts that synapses should be more readily depressed.

The most direct test of the shifted STDP hypothesis would be to observe the effect of almost synchronous pre- and postsynaptic spikes on synaptic strength. However, the results of such experiments could be difficult to interpret because of confounding factors such as the physiological delays mentioned above. For example, if the pre- and postsynaptic spikes are induced exactly at the same time, the timing of their arrival at the synapse is not necessarily synchronous. If a shift in the STDP window function acts as a stabilizing mechanism, synapses should get depressed when postsynaptic spikes are generated by presynaptic spikes with short latency. Therefore, as an alternative experiment we suggest inducing spikes only in the presynaptic neuron and allowing the postsynaptic firing to be affected by this presynaptic activity. One possible way to perform such an experiment is to hold the voltage of the postsynaptic neuron close to its firing threshold, so that individual EPSPs can induce a postsynaptic spike. In this case, if there is a stabilizing shift in the STDP window, strong synapses that induce short-latency postsynaptic action potentials abruptly should get depressed.

Shifted STDP results in a unimodal distribution of synaptic strengths. This finding is in agreement with the measurements of quantal synaptic currents [6], [7] and from paired recordings [8]. However, the observed distribution of peak EPSP amplitudes has a heavier tail than the gamma distribution obtained from shifted STDP (see also [36], [41]). STDP is unlikely to be the only mechanisms involved in shaping the distribution of synaptic strengths. Nevertheless, figure 4 shows that in presence of correlated input, this distribution can be quite broad. Thus, in the context of shifted STDP, a heavy-tailed distribution may be a sign of multiple correlated subgroups of input spike trains.

The synapses in the model we considered were current-based, meaning that each excitatory or inhibitory input injects a current waveform to the neuron regardless of the value of its membrane potential. We have also studied an analogous model with conductance-based synapses, and this does not qualitatively change the reported results. These results show that the outcome of competition between correlated and uncorrelated spike trains with shifted STDP depends on the firing state of the postsynaptic neuron, which can be controlled by the rate of its inhibitory inputs. This allows for a dynamic switching between anti-Hebbian and Hebbian forms of plasticity, and it might be related to the role of local inhibitory interneurons in switching the activity-dependent development of visual cortical circuits during the critical period [42]. We also showed that a shifted version of the triplet model is stable and implements both Hebbian and anti-Hebbian competitions, as in the shifted pair-based model. It is worth noting that the suppression model [22], [23] is inherently stable without any shift and shows solely anti-Hebbian competition between correlated and uncorrelated inputs [43].

In conclusion, a slightly shifted STDP window stabilizes synaptic strength, buffers firing rates, and can implement different modes of synaptic competition. The required shift may arise from properties of the NMDA receptor, or from random jitter. In light of their importance in determined the outcome of synaptic plasticity, we argue that the properties of STDP for short pairing intervals, which have not yet been clearly resolved, warrant a more detailed investigation.

## Methods

### Neural and synaptic models

The membrane potential of the integrate-and-fire model neuron obeys
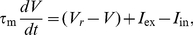
(3)where 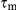 is the membrane time constant, 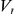 is the resting potential, 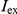 is the excitatory input and 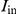 is the inhibitory input. Note that although these inputs appear as currents, they are actually measured in units of the membrane potential (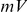) because a factor of the membrane resistance has been absorbed into their definition. When the membrane potential 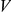 reaches the firing threshold 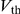, the neuron fires an action potential and the membrane potential resets to the resting value. The numerical values of all parameters are given in Table 1.

Each presynaptic action potential at an excitatory or inhibitory synapse induces an abrupt jump into the corresponding synaptic input (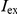 or 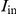), which decays exponentially afterwards. The time course of the synaptic inputs can thus be expressed as
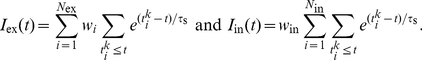
(4)Here, the first sums run over all excitatory (inhibitory) synapses (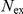 or 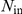, respectively). The second sums run over all the presynaptic spike times 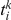, indexed by 
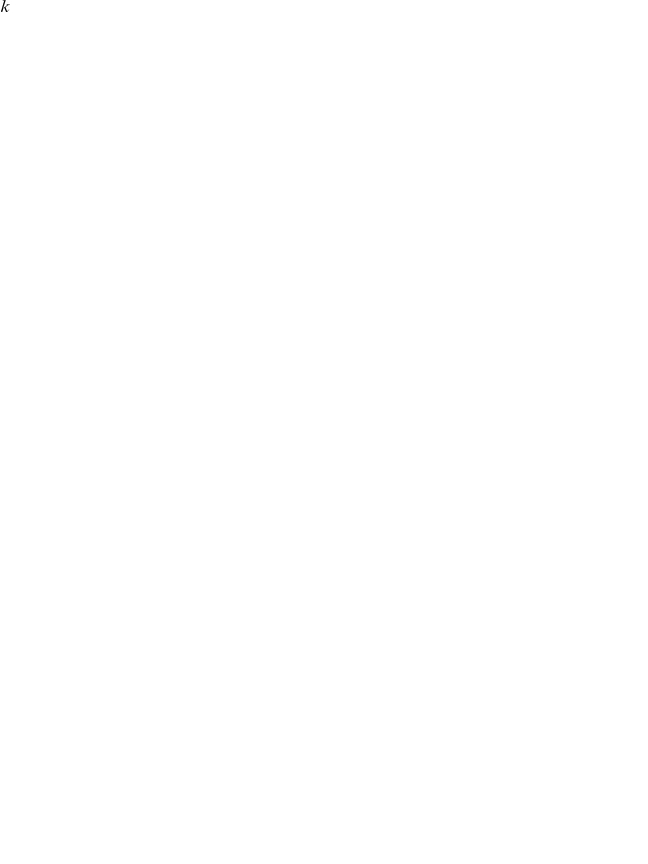
, reaching synapse 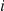 before time 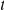. The synaptic time constant 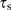 is taken to be the same for excitatory and inhibitory synapses. The inhibitory synaptic strength 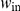 is fixed and is the same for all inhibitory synapses. The excitatory synaptic strengths 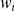 change due to STDP.

In all simulations, the synaptic strengths were initialized randomly from a uniform distribution over the range 1–5 mV. For each parameter regime, the simulations were run for 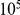 seconds of simulated time. The steady-state nature of the synaptic strengths was established when the first, second, and third moments of the distribution, as well as the average firing rate of the neuron, remained constant.

### Shifted triplet model

The triplet model [12] includes a presynaptic detector for each synapse 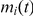 and a single postsynaptic detector 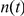. In the absence of incoming presynaptic spikes to synapse 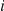, the value of the detector 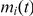 decays exponentially with the time constant 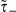. Likewise, the value of the postsynaptic detector 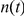 decreases exponentially in the absence of postsynaptic spikes with the time constant 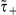. When a presynaptic spike reaches synapse 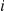 at time 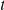, 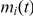 is set to 1, and 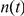 is set to 1 if there is a postsynaptic spike at time 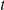. Formally,
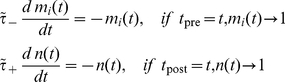
(5)For each excitatory synapse 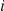, the change in synaptic strength, 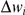, induced by a pair of pre- and postsynaptic action potentials with time difference 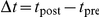 is determined by
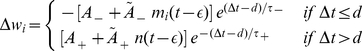
(6)The infinitesimally small parameter 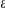 ensures that the values of 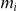 and 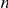 before their update by the immediate pre- or postsynaptic spikes are used.

### Correlated spike trains

To study synaptic competition, half of the excitatory input spike trains were correlated. To generate Poisson spike trains with homogeneous pairwise (zero-lag) correlations, we used the method developed by Kuhn et al [44]. First, a “generating” Poisson spike train with rate 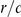 was produced. The correlated spike trains were then obtained by thinning the generating spike train, i.e. by randomly deleting spikes with probability 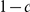. The resulting spike trains all have rate 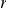, with each pair having the correlation coefficient 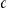. To introduce a non-zero lag to the spike trains, a random value drawn from an exponential distribution is added to each spike time. The mean of the exponential distribution is the correlation time constant.

### Derivation of the steady-state distribution of weights

The evolution of the distribution of synaptic strengths is described by the Fokker-Planck equation [14]–[16].
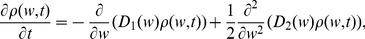
(7)where 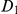 and 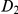 are drift and diffusion terms, respectively. To derive equilibrium distributions of synaptic strengths, we need the steady-state solution, obtained by setting the right side of equation 7 to zero. Solving the resulting ordinary differential equation for 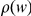, we obtain
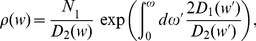
(8)where, 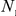 is a normalization constant.

The terms 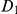 and 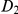 can be written as
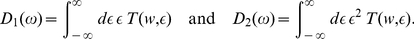
(9)Here, 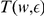 is the probability density of a synaptic modification that changes the strength of a given synapse from 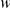 to 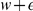.

When the synaptic strengths are changing due to STDP, the only relevant stochastic variable is the interval between the pre- and postsynaptic spike pairs. If a pairing of pre- and postsynaptic spikes occurs with interval 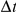, then 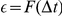, where 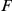 is the STDP window function (equation 1). To simplify the notation, we use 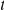 to denote 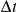 in the following equations. If the probability density of a pairing interval 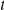 is 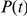, then the transitional probability density can be written as
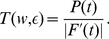
(10)With the transformations 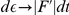 and 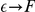, the terms 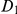 and 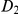 can be re-expressed as
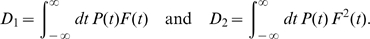
(11)Thus, to determine 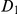 and 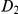 in terms of the parameters of the model, we only need to know the probability density of pairing intervals 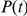.

We approximate the spiking behavior of the integrate-and-fire neuron by that of a linear Poisson neuron firing at the same rate [11], [15]. We first consider the case that the presynaptic spike follows the postsynaptic spike (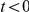). In this case, the timing of the presynaptic spike has no causal effect on the postsynaptic spike time. If we assume that both the presynaptic and postsynaptic spike trains are Poisson, the probability density of nearest-neighbor pairing intervals is
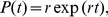
(12)where 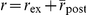 is the sum of the excitatory presynaptic firing rate (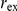) and the steady-state postsynaptic firing rate (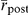). For an integrate-and-fire neuron, the steady-state firing rate can be approximated as
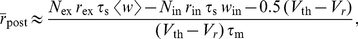
(13)where 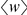 is the mean of the excitatory synaptic strengths. Now consider the case in which the postsynaptic spike follows a presynaptic spike (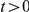). In this case, the arrival of the presynaptic spike increases the postsynaptic firing rate transiently. For an integrate-and-fire neuron, the instantaneous firing rate upon arrival of a presynaptic spike can be approximated as
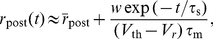
(14)where 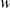 is the strength of the synapse at which the presynaptic spike arrived. The second term in equation 14 accounts for the correlation between pre- and postsynaptic spikes as calculated by Gütig et al [11] for a linear Poisson neuron, except that we have a synaptic time constant 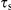. If we assume that both the presynaptic and postsynaptic spike trains are Poisson, the probability density of pairing intervals is
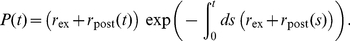
(15)If we assume that 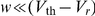, we can Taylor expand equation 15 to first order in 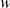 and, together with equation 14, the probability density of pairing intervals can be expressed as (see figure 1C & D)
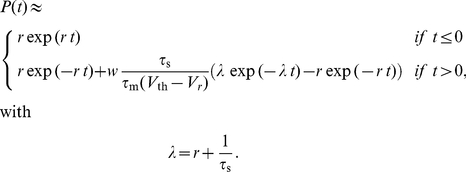
(16)Note that the second term in equation 16 for 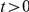 corresponds to the causal bump in figure 1C & D. The shape of the causal bump resembles that calculated by Cateau & Fukai [16] from the equation for the first passage time of a noisy integrate-and-fire neuron.

If we substitute 16 into equation 9, we obtain 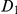 and 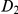 in terms of the parameters of the model. Because 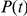 is linear in 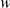, 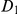 and 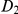 are also linear and can be written as
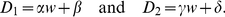
(17)Assuming that 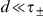, these coefficients can be written as
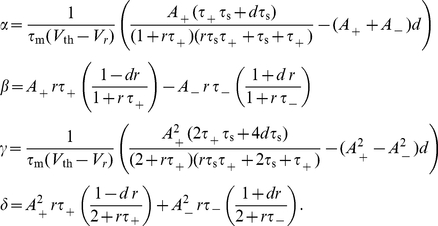
(18)


Finally, by inserting equations 17 into equation 8, we obtain the steady-state distribution
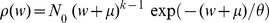
(19)with
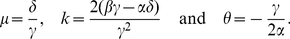
(20)Equation 19 is the same as equation 2 of the Results.

For the above distribution 19 to be normalizable, 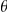 and 
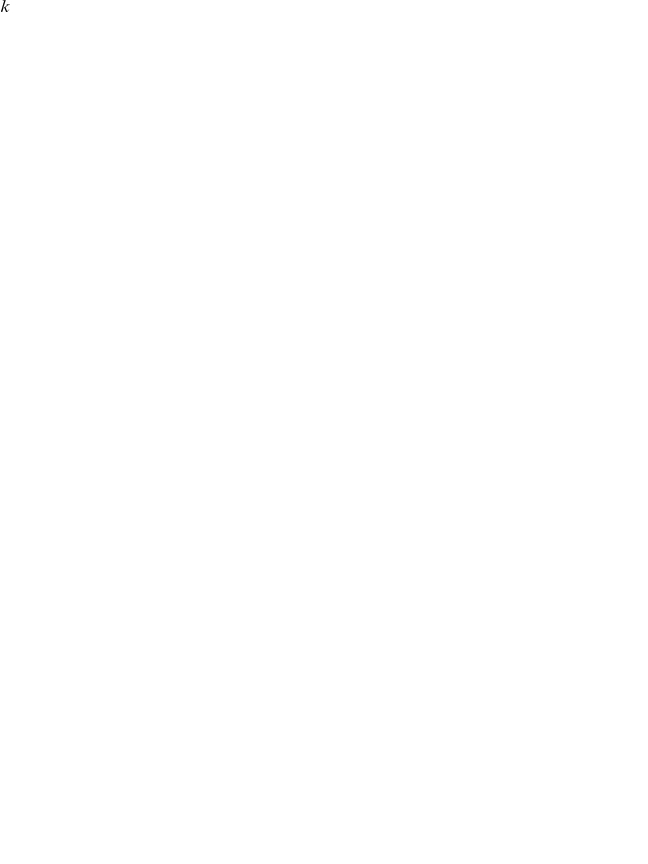
 must be positive. Equations 20 indicate that these conditions are met if 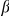, 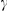 and 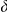, as given by equations 18, are all positive and 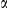 is negative. Provided that 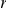 is less than of order 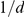 (which it always is at steady-state), 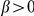 if 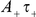 is sufficiently greater than 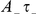, which is the condition stated in the text. Over the range we consider, 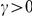 and 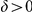 without requiring any further conditions. When 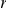 is greater than of order 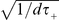, which it is at steady state, 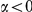, so stability is achieved.

If 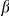 is positive, the mean synaptic strength is approximately
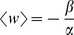
(21)Solving the above equation simultaneously with equation 13, gives the steady-state firing rate of the neuron, as depicted in figure 3 (inset). Having solved for the steady-state postsynaptic firing rate and the mean synaptic strength, the parameters of the distribution (equation 2) are fully expressed in terms of the model parameters.

## References

[1] Miller K (1996). Synaptic economics: Competition and cooperation in correlation-based synaptic plasticity.. Neuron.

[2] Bi G, Poo M (2001). Synaptic modification by correlated activity: Hebb's postulate revisited.. Annu Rev Neurosci.

[3] Caporale N, Dan Y (2008). Spike timing-dependent plasticity: a hebbian learning rule.. Annu Rev Neurosci.

[4] Dan Y, Poo M (2006). Spike timing-dependent plasticity: From synapse to perception.. Physiol Rev.

[5] Song S, Miller K, Abbott L (2000). Competitive hebbian learning through spike-timing-dependent synaptic plasticity.. Nat Neurosci.

[6] Turrigiano G, Leslie K, Desai N, Rutherford L, Nelson S (1998). Activity-dependent scaling of quantal amplitude in neocortical neurons.. Nature.

[7] O'Brien R, Kamboj S, Ehlers M, Rosen K, Fischbach G (1998). Activity-dependent modulation of synaptic ampa receptor accumulation.. Neuron.

[8] Song S, Sjostrom P, Reigl M, Nelson S, Chklovskii D (2005). Highly nonrandom features of synaptic connectivity in local cortical circuits.. PLoS Biol.

[9] van Rossum M, Bi G, Turrigiano G (2000). Stable hebbian learning from spike timing-dependent plasticity.. J Neurosci.

[10] Rubin J, Lee D, Sompolinsky H (2001). Equilibrium properties of temporally asymmetric hebbian plasticity.. Physical Review Letters.

[11] Gütig R, Aharonov R, Rotter S, Sompolinsky H (2003). Learning input correlations through nonlinear temporally asymmetric hebbian plasticity.. J Neurosci.

[12] Pfister J, Gerstner W (2006). Triplets of spikes in a model of spike timing-dependent plasticity.. J Neurosci.

[13] Sjöström P, Turrigiano G, Nelson S (2001). Rate, timing, and cooperativity jointly determine cortical synaptic plasticity.. Neuron.

[14] Risken H (1996). The Fokker-Planck equation.

[15] Kempter R, Gerstner W, van Hemmen J (2001). Intrinsic stabilization of output rates by spike-based hebbian learning.. Neural Comput.

[16] Cateau H, Fukai T (2003). A stochastic method to predict the consequence of arbitrary forms of spike-timing-dependent plasticity.. Neural Comput.

[17] Gerstein GL, Mandelbrot B (2006). Random walk models for the spike activity of a single neuron.. Biophys J.

[18] Shadlen M, Newsome W (1998). The variable discharge of cortical neurons: implications for connectivity, computation, and information coding.. J Neurosci.

[19] Troyer T, Miller K (1997). Physiological gain leads to high isi variability in a simple model of a cortical regular spiking cell.. Neural Comput.

[20] Salinas E, Sejnowski T (2000). Impact of correlated synaptic input on output firing rate and variability in simple neuronal models.. J Neurosci.

[21] Froemke R, Poo M, Dan Y (2005). Spike-timing-dependent synaptic plasticity depends on dendritic location.. Nature.

[22] Froemke R, Tsay I, Raad M, Long J, Dan Y (2006). Contribution of individual spikes in burst-induced long-term synaptic modification.. J Neurophysiol.

[23] Froemke RC, Dan Y (2002). Spike-timing-dependent synaptic modification induced by natural spike trains.. Nature.

[24] Wang HX, Gerkin RC, Nauen DW, Bi GQ (2005). Coactivation and timing-dependent integration of synaptic potentiation and depression.. Nat Neurosci.

[25] Bienenstock E, Cooper L, Munro P (1982). Theory for the development of neuron selectivity: orientation specificity and binocular interaction in visual cortex.. J Neurosci.

[26] Sjöström P, Turrigiano G, Nelson S (2003). Neocortical ltd via coincident activation of presynaptic nmda and cannabinoid receptors.. Neuron.

[27] Bender V, Bender K, Brasier D, Feldman D (2006). Two coincidence detectors for spike timing-dependent plasticity in somatosensory cortex.. J Neurosci.

[28] Malenka R, Bear M (2004). Ltp and ltd:: An embarrassment of riches.. Neuron.

[29] Nowak L, Bregestovski P, Ascher P, Herbet A, Prochiantz A (1984). Magnesium gates glutamate-activated channels in mouse central neurones.. Nature.

[30] Jahr C, Stevens C (1990). A quantitative description of nmda receptor-channel kinetic behavior.. J Neurosci.

[31] Destexhe A, Mainen Z, Sejnowski T (1994). Synthesis of models for excitable membranes, synaptic transmission and neuromodulation using a common kinetic formalism.. J Comput Neurosci.

[32] Shouval H, Bear M, Cooper L (2002). A unified model of nmda receptor-dependent bidirectional synaptic plasticity.. Proc Natl Acad Sci U S A.

[33] Karmarkar UR, Najarian MT, Buonomano DV (2002). Mechanisms and significance of spike-timing dependent plasticity.. Biol Cybern.

[34] Lubenov E, Siapas A (2008). Decoupling through synchrony in neuronal circuits with propagation delays.. Neuron.

[35] Magee J, Cook E (2000). Somatic epsp amplitude is independent of synapse location in hippocampal pyramidal neurons.. Nat Neurosci.

[36] Andrasfalvy B, Magee J (2001). Distance-dependent increase in ampa receptor number in the dendrites of adult hippocampal ca1 pyramidal neurons.. J Neurosci.

[37] Rumsey C, Abbott L (2004). Equalization of synaptic efficacy by activity-and timing-dependent synaptic plasticity.. J Neurophysiol.

[38] Gidon A, Segev I (2009). Spike-timing-dependent synaptic plasticity and synaptic democracy in dendrites.. J Neurophysiol.

[39] Sjöström P, Häusser M (2006). A cooperative switch determines the sign of synaptic plasticity in distal dendrites of neocortical pyramidal neurons.. Neuron.

[40] Letzkus JJ, Kampa BM, Stuart GJ (2006). Learning rules for spike timing-dependent plasticity depend on dendritic synapse location.. J Neurosci.

[41] Katz Y, Menon V, Nicholson DA, Geinisman Y, Kath WL (2009). Synapse distribution suggests a two-stage model of dendritic integration in ca1 pyramidal neurons.. Neuron.

[42] Hensch T (2005). Critical period plasticity in local cortical circuits.. Nat Rev Neurosci.

[43] Babadi B, Abbott LF (2010). Stability and competition in multi-spike models of spike-timing dependent plasticity..

[44] Kuhn A, Aertsen A, Rotter S (2003). Higher-order statistics of input ensembles and the response of simple model neurons.. Neural Comput.

